# Dengue Virus Genome Uncoating Requires Ubiquitination

**DOI:** 10.1128/mBio.00804-16

**Published:** 2016-06-28

**Authors:** Laura A. Byk, Néstor G. Iglesias, Federico A. De Maio, Leopoldo G. Gebhard, Mario Rossi, Andrea V. Gamarnik

**Affiliations:** a Fundación Instituto Leloir, CONICET, Buenos Aires, Argentina; b Instituto de Investigación en Biomedicina de Buenos Aires (IBioBA)-CONICET-Partner Institute of the Max Planck Society, Polo Científico Tecnológico, Buenos Aires, Argentina

## Abstract

The process of genome release or uncoating after viral entry is one of the least-studied steps in the flavivirus life cycle. Flaviviruses are mainly arthropod-borne viruses, including emerging and reemerging pathogens such as dengue, Zika, and West Nile viruses. Currently, dengue virus is one of the most significant human viral pathogens transmitted by mosquitoes and is responsible for about 390 million infections every year around the world. Here, we examined for the first time molecular aspects of dengue virus genome uncoating. We followed the fate of the capsid protein and RNA genome early during infection and found that capsid is degraded after viral internalization by the host ubiquitin-proteasome system. However, proteasome activity and capsid degradation were not necessary to free the genome for initial viral translation. Unexpectedly, genome uncoating was blocked by inhibiting ubiquitination. Using different assays to bypass entry and evaluate the first rounds of viral translation, a narrow window of time during infection that requires ubiquitination but not proteasome activity was identified. In this regard, ubiquitin E1-activating enzyme inhibition was sufficient to stabilize the incoming viral genome in the cytoplasm of infected cells, causing its retention in either endosomes or nucleocapsids. Our data support a model in which dengue virus genome uncoating requires a nondegradative ubiquitination step, providing new insights into this crucial but understudied viral process.

## IMPORTANCE

Dengue is the most significant arthropod-borne viral infection in humans. Although the number of cases increases every year, there are no approved therapeutics available for the treatment of dengue infection, and many basic aspects of the viral biology remain elusive. After entry, the viral membrane must fuse with the endosomal membrane to deliver the viral genome into the cytoplasm for translation and replication. A great deal of information has been obtained in the last decade regarding molecular aspects of the fusion step, but little is known about the events that follow this process, which leads to viral RNA release from the nucleocapsid. Here, we investigated the fate of nucleocapsid components (capsid protein and viral genome) during the infection process and found that capsid is degraded by the ubiquitin-proteasome system. However, in contrast to that observed for other RNA and DNA viruses, dengue virus capsid degradation was not responsible for genome uncoating. Interestingly, we found that dengue virus genome release requires a nondegradative ubiquitination step. These results provide the first insights into dengue virus uncoating and present new opportunities for antiviral intervention.

## INTRODUCTION

Dengue virus (DENV) belongs to the *Flavivirus* genus in the *Flaviviridae* family, together with other emerging and reemerging human pathogens that cause fever and encephalitis, such as Zika virus (ZIKV), West Nile virus (WNV), Japanese encephalitis virus (JEV), and Saint Louis encephalitis virus (SLEV).

DENV is the most prevalent mosquito-borne human viral pathogen worldwide, with around 3.6 billion people currently living in areas of endemicity. About 390 million infections in total and 100 million symptomatic cases of DENV infection are estimated to occur each year (1). In the Americas, a steady increase in the number of DENV infections is being recorded, and areas formerly considered DENV free are registering the first outbreaks of dengue fever due to the mobility of people from regions of endemicity and the presence of the *Aedes aegypti* vector (WHO; http://apps.who.int/iris/bitstream/10665/75303/1/9789241504034_eng.pdf?ua=1). Despite this great burden and the urgent medical need to control DENV infections, effective therapeutics are still unavailable.

DENV bears a plus-strand RNA genome of about 11 kb that includes a single open reading frame. The genome is translated into a single polyprotein that is cleaved into three structural proteins (capsid, envelope [E], and membrane [prM/M]) and at least seven nonstructural proteins (2). In addition to serving as a messenger for translation, the genome is a template for RNA replication and encapsidation. In the last decade, a great deal has been learned about molecular mechanisms that regulate viral genome replication (3). However, little is known about the processes that lead to genome encapsidation and uncoating. These two fundamental viral steps involve opposite functions of the viral capsid protein, which must recruit and release the genome during assembly and uncoating, respectively.

The capsid protein is a highly basic protein of 12 kDa that binds RNA with high affinity but low specificity. The protein forms homodimers in solution and oligomerizes upon nucleic acid binding. The tridimensional structure, solved by nuclear magnetic resonance (NMR) analysis, indicates the formation of 4 α-helices (α1 to α4) (4). Both the N-terminal region, unstructured in solution, and the α4 helix contain clusters of positive charges that are essential for RNA binding and particle assembly (5, 6). Viral particle reconstructions have suggested that the capsid works as a nucleoprotein that neutralizes and condenses the viral RNA without a defined structure (7, 8).

Recent studies have focused on capsid functions for drug development to control DENV infections (9–11). Although still little explored, the capsid is an attractive target for antivirals due to the multimeric assembly of the protein. On the basis of the concept of genetic dominance of defective subunits, oligomerization of capsid units, including both resistant and susceptible proteins, in infected cells would generate nonfunctional complexes, delaying emergence of resistant viruses (11).

In DENV-infected cells, capsid proteins distribute in different subcellular compartments. They accumulate in the nucleus (mainly in the nucleolus) and associate with lipid droplets (LDs) and endoplasmic reticulum (ER) membranes (12–17). The functional significance of the nuclear and LD accumulation of capsid is still unclear. Viral assembly takes place in close association with RNA replication complexes near ER membranes, where capsid proteins presumably interact with the viral RNA to form the nucleocapsid, which in turn buds into the ER, acquiring the lipid bilayer containing E and prM/M. The immature viral particles then travel through the secretory pathway and, after maturation by furin activity, are released.

During a new infection, the viral particle is internalized by receptor-mediated endocytosis and, upon endosome acidification, conformational changes in the E protein lead to membrane fusion (18, 19). This process results in the release of the viral nucleocapsid into the cytoplasm, where the genome is used for translation. How the capsid protein is removed from the viral RNA and how the genome is released to enable protein synthesis during infection are still obscure steps in the biology of DENV and other flaviviruses.

To examine the process of DENV genome uncoating, we analyzed the fate of capsid protein during infection. We describe here for the first time the kinetics of the incoming capsid and found that the protein is degraded after internalization by a ubiquitin-proteasome-dependent process. Although capsid degradation seemed to temporarily correlate with the initial rounds of viral translation, genome uncoating was found to be independent of the proteasome activity. Pharmacological inhibition of the proteasome was sufficient to increase capsid stability but did not impair nucleocapsid disassembly. Importantly, inhibition of the cellular E1 ubiquitin-activating enzyme was found to avoid the liberation of the incoming viral RNA during infection, impairing access of the viral genome to the translation machinery. Our data provide new insight into DENV uncoating and present new ideas for antiviral intervention.

## RESULTS

### Fate of the incoming DENV capsid protein during viral infection.

**FIG 1:**
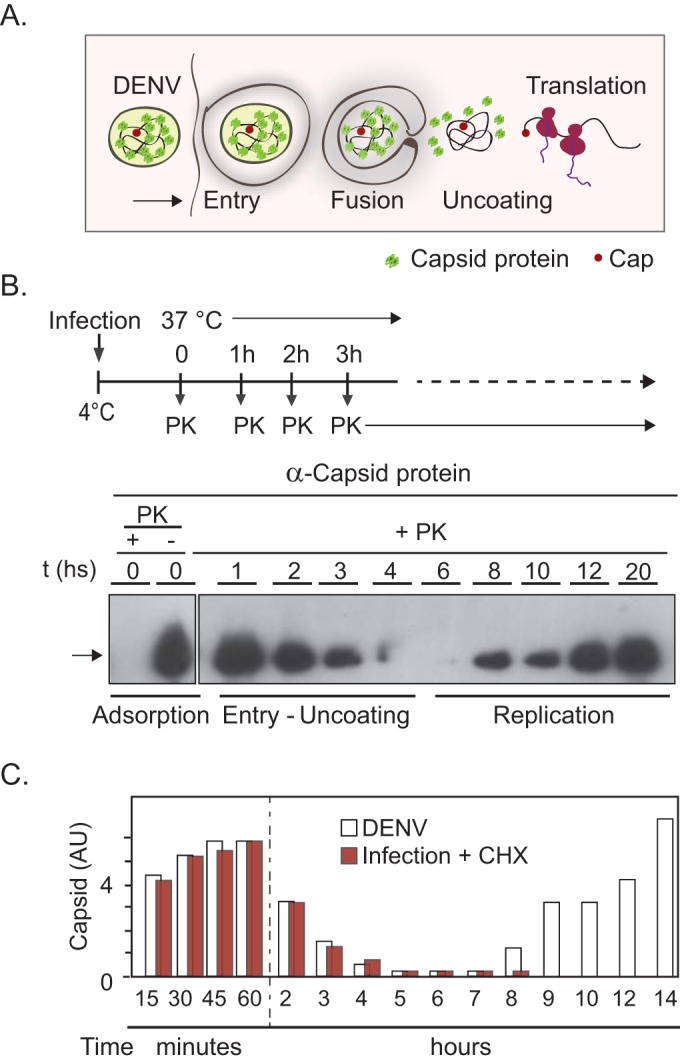
Fate of the capsid protein during DENV infection. (A) Schematic representation of the events during viral entry and uncoating. (B) (Top) Experimental design showing the times of infection (above time line) and times of sample collection (below time line). (Bottom) Western blots using DENV anticapsid antibodies are shown. Adsorption lanes indicate the capsid protein present in adsorbed virions in the absence of proteinase K (PK) treatment. In the presence of PK, complete removal of adsorbed virions was observed. The presence of the capsid protein during viral infection at different times from 1 to 20 h postinfection after PK treatment is shown. (C) Levels of capsid protein inside the host cell as a function of time postinfection in the presence (orange bars) and absence (white bars) of cycloheximide (CHX). AU, arbitrary units.

After DENV entry, the viral particle remains inside endosomes, and after endosome maturation and membrane fusion, the nucleocapsid is released into the cytoplasm (Fig. 1A). To study these initial steps that lead to genome uncoating during DENV infection, we first developed a protocol to detect the incoming capsid protein in adsorbed and internalized virions. We examined changes in capsid protein levels as a function of time after DENV2 infection of A549 cells by the use of Western blots. Capsids of adsorbed viruses were readily detected by inoculation at 4°C (Fig. 1B). To examine the internalized capsid protein, adsorbed viruses were removed by proteinase K (PK) treatment. Cells kept at 4°C and treated with PK showed complete removal of adsorbed viruses (Fig. 1B). After the temperature was shifted to 37°C, internalized viruses were analyzed as a function of time, and adsorbed (noninternalized) particles were removed at each time point by PK treatment. The amount of capsid in infecting virions was a limitation for protein detection; thus, scaling up the amount of infected cells was necessary for all the studies whose results are described below. This assay allowed not only detection of capsid protein that enters the cell during infection but also analysis of the kinetics of protein levels during the whole viral life cycle. A time course analysis postinfection showed the degradation of capsid protein, with an estimated half-life of 90 min. Capsid was barely detected after 4 h and remained undetectable until 8 h postinfection. At that time, an exponential increase in protein levels, representing protein that was presumably newly synthesized as a result of genome amplification, was observed (Fig. 1B).

It is possible that the first rounds of translation contributed to the first peak of capsid protein detected. To examine this possibility, we extended the time course to evaluate the levels of capsid inside infected cells, in the presence or absence of cycloheximide (CHX). The cellular translation is inhibited by the use of this compound, allowing the detection of only the incoming capsid protein. The levels of capsid detected between 15 min and 4 h post-DENV infection were not significantly different in the presence and absence of the translation inhibitor (Fig. 1C). The second peak of capsid accumulation was not detected in the presence of CHX due to inhibition of RNA replication in the absence of viral translation (Fig. 1C). This indicates that the translated capsid protein from the incoming viral genome does not significantly contribute to the amount of detected protein during the first hours postinfection. We conclude that capsid levels follow a biphasic curve during infection, with the first peak corresponding to capsid inside internalized virions and the second peak associated with newly synthesized protein from amplified genomic RNA.

### The incoming DENV capsid protein is subjected to proteasome-dependent degradation.

**FIG 2:**
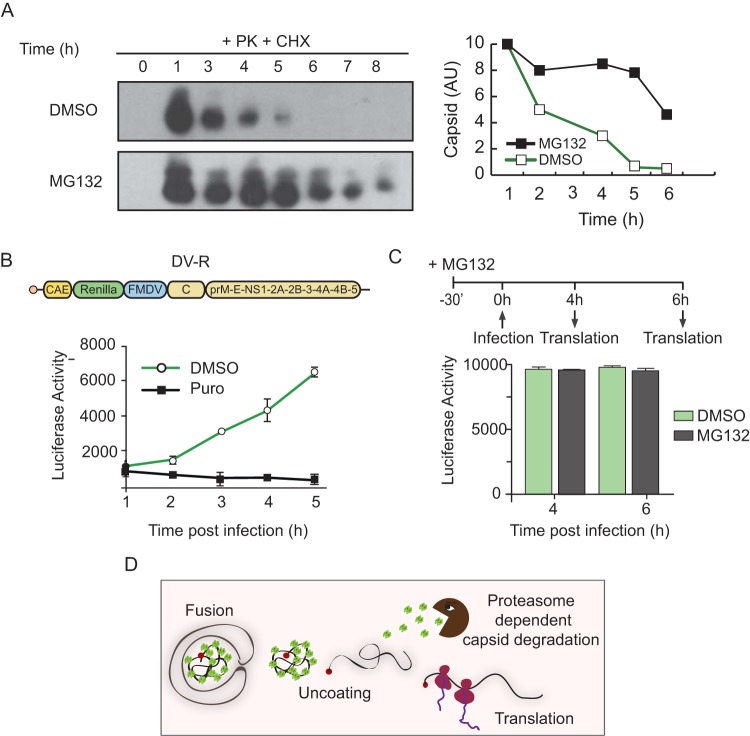
Capsid protein decay is proteasome dependent, but its degradation is not required for initial viral translation. (A) A representative Western blot showing viral capsid protein decay during infection in the presence of CHX and DMSO (top) or CHX and the MG132 proteasome inhibitor (bottom). On the right, a plot shows quantified capsid levels as a function of time in the presence of DMSO or MG132. (B) (Top panel) Schematic representation of a reporter dengue virus, DV-R, showing the duplication of the *cis*-acting elements (CAE) and the location of luciferase and viral protein-coding regions. FMDV, foot-and-mouth desease virus 2A site; C, capsid. (Bottom panel) Measurement of the first rounds of viral translation by analysis of luciferase activity in the presence (black line) or absence (green line) of puromycin (Puro). (C) Luciferase activity measurement in cells infected with DV-R in the presence (gray bars) or absence (green bars) of MG132 at 4 or 6 h postinfection. Data correspond to the averages of results of three experiments. Error bars indicate standard deviations. (D) Cartoon representing the results obtained where capsid degradation was not necessary for the first rounds of viral translation.

To examine whether capsid degradation is proteasome dependent, we used a protocol similar to the one described above but included the use of the proteasome inhibitor MG132. Cells were infected with DENV2 and treated with CHX to avoid detection of newly synthesized protein in the presence or absence of MG132. A time course analysis of capsid levels under these conditions showed a significant delay in protein degradation, suggesting a proteasome-dependent process. We observed that the half-life of the incoming capsid increased from about 90 min to 5 h in the presence of MG132 (Fig. 2A). On the basis of this observation, we hypothesized that DENV nucleocapsid disassembly requires proteasome activity for capsid degradation, similarly to that described for poxviruses (20).

An important method to assess genome release in the cytoplasm is measurement of the first rounds of translation, which were undetectable by Western blot analysis of capsid or other viral proteins tested (Fig. 1C). Thus, we evaluated whether a reporter virus carrying a luciferase gene was amenable to detection of early translation of the incoming viral RNA. To this end, we used a reporter system carrying the full-length viral genome fused to *Renilla* luciferase that we had previously developed in our laboratory (dengue virus R [DV-R]) (14). Infection with DV-R allowed detection of translation, as distinguished from that occurring in the presence of puromycin, as early as 2 h postinfection (Fig. 2B).

If capsid degradation is necessary for genome uncoating, the first rounds of translation would be impaired by proteasome inhibition. Thus, we examined the impact of MG132 on translation of the incoming genome using the DV-R system. Unexpectedly, the viral RNA was readily available for translation in the presence of the inhibitor. The measurements of translation at 4 and 6 h postinfection were similar in the presence and absence of MG132 (Fig. 2C). The results support the idea that the incoming capsid protein is degraded by a proteasome-dependent process; however, this event is not required for genome release. We cannot rule out the possibility of an active role of the ribosomes in displacing capsid from the RNA (21), but it is possible to conclude that capsid degradation is not the cause of DENV genome uncoating and that protein decay likely takes place when the RNA is already engaged in translation (Fig. 2D).

### Ubiquitination is required for the initial rounds of DENV translation.

**FIG 3:**
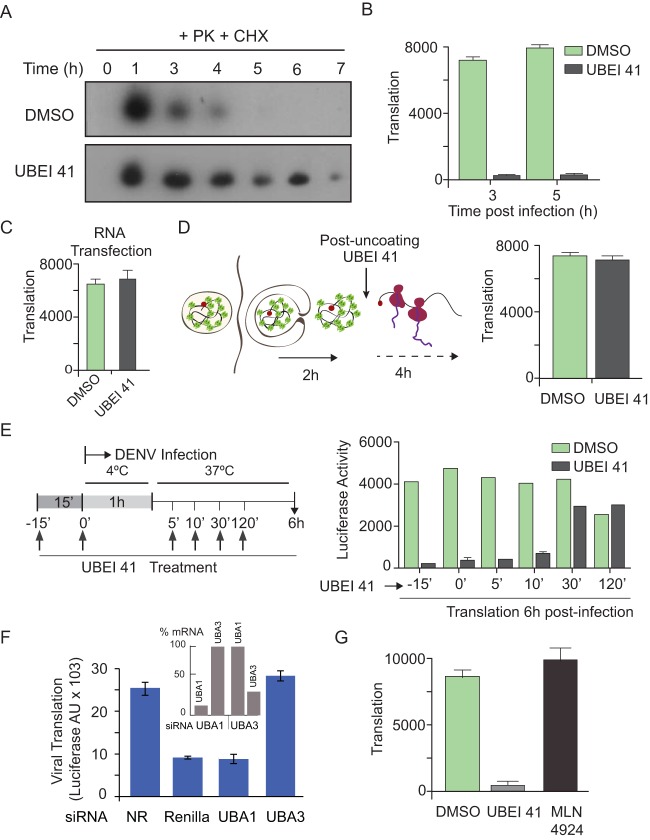
Capsid degradation and viral translation depend on ubiquitination. (A) A representative Western blot showing viral capsid protein decay during infection in the presence of CHX and DMSO (top) or CHX and the ubiquitination inhibitor UBEI 41 (bottom). (B) Luciferase activity measurement in cells infected with DV-R in the presence (gray bars) or absence (green bars) of UBEI 41 at 3 and 5 h postinfection. (C) Luciferase activity measurement in cells transfected with DENV reporter RNA to bypass infection, in the presence or absence of UBEI 41. (D) (Left panel) Schematic representation of the experimental design: after 2 h of infection with DV-R, UBEI 41 (or DMSO [control]) was added; luciferase activity was measured 4 h after addition of the compound. (Right panel) Luciferase levels in the presence or absence of UBEI 41 as indicated. (E) (Left panel) Experimental design showing the times of infection, UBEI 41 addition, and sample collection. (Right panel) Luciferase activity measurement in cells infected with DENV reporter in the presence or absence of UBEI 41 at 6 h postinfection. Data correspond to the averages of results of three experiments. (F) DENV reporter infections of cells silenced with siRNAs directed to UBA1 (ubiquitin-like modifier activating enzyme 1; required for ubiquitination), UBA3 (ubiquitin-like modifier activating enzyme 3; required for neddylation), *Renilla* (positive control), or a nonrelated siRNA (NR [negative control]). AU X 103, AU × 10^3^. (Inset panel) Quantification of mRNA levels of UBA1 or UBA3 in cells silenced for one or the other gene by real-time RT-PCR. (G) Luciferase activity measurement in cells infected with reporter DENV in the presence of UBEI 41, DMSO, or the MLN4924 neddylation inhibitor as indicated at 6 h postinfection.

Considering that the proteasome pathway is typically ubiquitin dependent, we further evaluated whether ubiquitination was necessary for incoming viral capsid degradation. To this end, we examined whether inhibition of the ubiquitination machinery increased capsid stability during infection. UBEI 41, an inhibitor of ubiquitin-like modifier activating enzyme 1 (UBA1), was used. DENV-infected cells were incubated in the presence of CHX to avoid detection of newly synthetized protein at late time points and were treated with UBEI 41 or left untreated. Cell extracts were taken every hour and treated with PK as previously described. A slight delay in viral entry was observed, as the amount of capsid internalized was about 20% less than that detected in the presence of dimethyl sulfoxide (DMSO) (control). Importantly, a great impact on the stability of the internalized capsid was observed in the presence of UBEI 41 (Fig. 3A). The half-life of capsid changed from 90 min to about 6 h. The delay in protein degradation was similar to that observed upon proteasome inhibition. These results support the idea of a role of the ubiquitin-proteasome pathway in viral capsid decay during DENV infection.

Then, we attempted to confirm that genome uncoating was also independent of ubiquitination. Therefore, we used the reporter virus to measure early translation events. Cells were infected with DV-R in the presence of UBEI 41 or DMSO, and translation was analyzed as a function of time. Surprisingly, a remarkable inhibition of viral translation was observed in the presence of the ubiquitin E1 inhibitor (Fig. 3B). On the basis of this result, it was possible that UBEI 41 exerted a direct effect on viral translation. Thus, we bypassed the entry-uncoating step by transfecting the viral RNA and translation was examined in the presence or absence of UBEI 41. Under these conditions, translation was unaffected by E1 inhibition (Fig. 3C). To confirm this result, we designed an additional control experiment in which viral infection was performed in the absence of UBEI 41, viral internalization and uncoating were allowed for 2 h, and the inhibitor was then added (Fig. 3D, left panel). These infected cells were subsequently used to evaluate the impact of the inhibitor directly on translation 6 h postinfection. Under these conditions, the translation levels of cells treated with UBEI 41 or DMSO were indistinguishable (Fig. 3D). Together, the results confirm that the ubiquitin E1 enzyme activity is necessary during an early step in DENV infection, after internalization but previous to viral translation. Interestingly, this requirement was found to be independent of the proteasome activity (Fig. 2C).

To better define the window of sensitivity to UBEI 41, we performed a time course, preincubating or adding the inhibitor, or DMSO, at different times postinfection (5, 10, 30, and 120 min) (Fig. 3E, left panel). An evident reduction of the effect of the drug was observed when the inhibitor was added 30 min after infection, suggesting that the ubiquitination machinery is necessary early during DENV infection (Fig. 3E). Because UBEI 41 does not impair viral entry (Fig. 3A) or translation (Fig. 3D), we identified a sensitive step during genome uncoating (postentry and pretranslation) for DENV inhibition.

To further confirm the requirement of E1 activity for a productive infection, we silenced the protein target of UBEI 41 (UBA1) using small interfering RNAs (siRNAs). Cells were transfected with positive and negative siRNA controls or directed to UBA1. After confirming a reduction of about 85% of the levels of UBA1 mRNA at 48 h posttransfection, cells were infected with DV-R, and translation was measured at early time points to evaluate the first rounds of translation. Under these conditions, silencing UBA1 resulted in a 4-fold inhibition of viral translation similar to that observed for the *Renilla* luciferase siRNA positive control (Fig. 3F). The data confirm the requirement of E1 for an early event during DENV infection.

The requirement of ubiquitination of proteins that participate in maturation and trafficking of endosomes has been previously described (22–24). For different viruses, including influenza and vaccinia viruses, it has been shown that members of the large family of cullin-RING E3 ligases (CRL), such as Cullin3, are involved in viral uncoating (20, 25). To examine whether this requirement was common for DENV, we silenced a protein that has been shown to be essential for the activity of these E3 ligases (reviewed in reference 26) that neddylates the members of the CRL family. In this regard, siRNAs directed to UBA3, a component of the NEDD8 E1-activating enzyme, were used to assess the impact on DENV entry and translation. The results indicated that, in contrast to that observed by silencing UBA1, downregulation of UBA3 did not affect the first rounds of DENV translation (Fig. 3F). To confirm this observation by an alternative method, a pharmacological inhibitor of neddylation, MLN 4924, which has been reported to block the activity of members of the CRL family of E3 ligases, was also used. In this experiment, cells were treated with MLN 4924, UBEI 41, or DMSO and infected with DV-R. Translation was unaffected by MLN 4924, further suggesting that DENV does not require any CRL activity for genome uncoating (Fig. 3G).

### DENV containing a K-less capsid protein is competent for uncoating.

**FIG 4:**
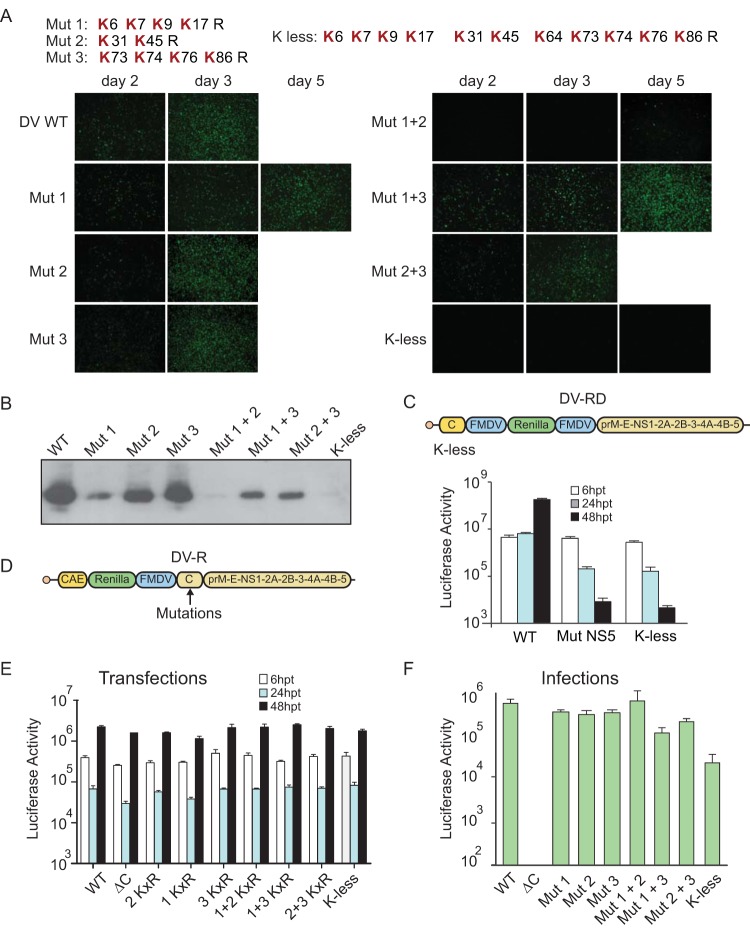
Infectivity of recombinant DENVs with substitution of lysine (K) by arginine (R) in the capsid protein. (A) (Top panel) Location of the capsid K residues that were substituted in the mutant viruses (Mut 1, Mut 2, and Mut 3). Combined mutations (Mut 1 plus Mut 2 [Mut 1+2], Mut 1+3, Mut 2+3, and K-less) were also designed. (Bottom panel) Results of immunofluorescence (IF) assays of cells transfected with WT or mutated viral RNAs at different times as indicated for each panel. (B) Expression and processing of capsid was evaluated by Western blot analysis after RNA transfection of the WT and each mutant: Mut 1, Mut 2, Mut 3, Mut 1+2, Mut 1+3, Mut 2+3, or K-less. (C) (Top panel) Schematic representation of a DENV reporter system, DV-RD, indicating the locations where the K substitutions were incorporated. (Bottom panel) Translation and replication of viral RNAs in reporter-transfected cells followed by luciferase activity measurements. A WT virus, a K-less mutant, and a replication impaired control virus (Mut NS5 [polymerase mutant]) are shown. hpt, hours posttransfection. (D) Schematic representation of the reporter dengue virus, DV-R, showing the locations of the mutations inserted in the capsid protein. (E) Translation and replication of viral RNAs corresponding to WT DV-R, a capsid-deleted control (ΔC), and the lysine mutants. (F) Evaluation of production of infectious particles. Levels of luciferase activity measured postinfection with supernatants obtained from the transfections presented in panel E.

We found that capsid protein is degraded by the proteasome but that this process is not required for genome release into the cytoplasm. In addition, ubiquitin E1 activity was found to be necessary for translation of the incoming viral genome but not for viral internalization. It is possible that capsid ubiquitination functions as a signal for nucleocapsid disassembly, as it was previously described for the disassembly of cellular ribonucleoprotein complexes (27). To evaluate whether capsid requires a nondegradative ubiquitination step for RNA release during infection, we generated recombinant DENVs in which the 11 Lys residues in capsid (possible targets for ubiquitination) were partially or completely replaced by Arg. Six recombinant viruses with partial K×R replacement, and one virus carrying only Arg (K-less capsid) were designed. (Fig. 4A, top panel). Cells were transfected with viral RNAs corresponding to these mutants along with a parental (wild-type [WT]) virus RNA, and viral propagation was examined by immunofluorescence (IF). Mutant 2 (Mut 2) and Mut 3 propagated similarly to the WT, while Mut2 plus Mut3 (Mut2+3) showed a slight delay in propagation. In contrast, all the genomes of the mutants that included the substitution introduced into Mut 1 (Mut 1, Mut 1+2, Mut 1+3, and K-Less) showed either a pronounced delay of or an impairment in viral propagation (Fig. 4A). To further evaluate viral infection of these mutants, we examined the level of expression of capsid protein for each case by Western blot analysis (Fig. 4B). The results of this analysis correlated with those of the IF assay. There are several possible explanations of these results. It is possible that Lys residues at specific positions are necessary for DENV assembly and/or infection, including possible ubiquitination; however, because the capsid coding sequence contains *cis*-acting RNA elements, an impact of the substitutions on RNA structures and viral RNA synthesis is also possible.

To evaluate this last possibility, the KxR mutations were introduced into a DENV reporter system, DV-RD (Fig. 4C, top panel). RNA corresponding to the DV-RD K-less was transfected into A549 cells together with two controls, the WT RNA and a replication impaired with a mutated polymerase (Mut NS5). The levels of translation of the three RNAs were indistinguishable; however, RNA synthesis of the DV-RD K-less measured at 24 and 48 h posttransfection was impaired (Fig. 4C), indicating that the introduced nucleotide changes drastically affected genome replication. These findings will be followed up in a separate study.

To evaluate the impact of the mutations exclusively on capsid protein functions, we used a different reporter virus which contains a duplication of the *cis*-acting RNA elements and introduced all the mutations in the coding sequence of capsid protein (Fig. 4D). Transfection of the mutated viral RNAs indicated that translation and RNA replication were efficient, confirming the successful uncoupling of RNA replication and capsid functions (Fig. 4E). To evaluate newly generated viral particles and to test their infectivity, the medium from transfected cells was used to infect fresh cells and luciferase activity was evaluated. If Lys ubiquitination is a requirement for viral uncoating, the K-less virus would not be infectious. Although Mut 1+3 and the K-less viruses showed some reduction in infectivity, they retained the ability to produce particles and to infect new cells. In addition, the other mutants showed infection levels similar to those seen with the WT virus (Fig. 4F). Together, the results suggest that genome uncoating does not depend on Lys ubiquitination of capsid. The possibility of capsid ubiquitination at noncanonical residues cannot be excluded.

### Ubiquitination inhibition blocks DENV genome release during infection.

**FIG 5:**
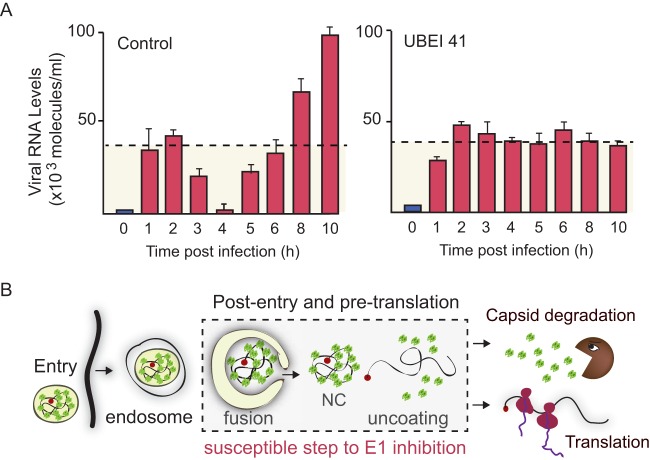
Ubiquitination inhibition blocks viral genome release during infection. (A) Levels of viral RNA measured by real-time RT-PCR as a function of time postinfection of cells treated with DMSO (left panel [Control]) or UBEI 41 (right panel). Adsorbed viral particles were removed with PK treatment at all time points. Data correspond to the averages of results of triplicates, and error bars indicate standard deviations. (B) Cartoon representing the steps of the viral life cycle that are susceptible to ubiquitination inhibition.

To further study the requirement of ubiquitination for viral uncoating, we examined the fate of the incoming viral genome during DENV infection and evaluated the impact of UBA1 inhibition. Viral RNA was quantified during the complete viral life cycle. The pattern of RNA levels as a function of time postinfection was strikingly similar to that observed for capsid protein (compare Fig. 5A with Fig. 1C). The highest level of incoming genome was detected between 1 and 2 h postinfection, and then the level dropped. Taking into account the translation kinetics, the incoming genome was uncoated, translated, and mostly degraded within a few hours after entry. Less than 5% of the incoming genome remained at 4 h postinfection. This RNA serves as the substrate for RNA amplification, which leads to an exponential increase in RNA levels (Fig. 5A, left panel). To evaluate the impact of ubiquitin E1 inhibition on the fate of the viral RNA, cells treated with UBEI 41 were infected and RNA levels were examined as described above. Interestingly, the viral RNA was internalized under these conditions, but levels remained the same even at 10 h postinfection (Fig. 5A, right panel). This result indicates that genome uncoating/degradation is avoided by inhibition of E1 activity. The data confirm the requirement of a ubiquitination step for genome release either from endosomes or nucleocapsids and explain why the first rounds of viral translation were impaired in the absence of ubiquitination (Fig. 5B).

## DISCUSSION

The mechanism by which the viral genome is released from the nucleocapsid during infection is one of the least-characterized processes of the flavivirus life cycles. Here, we determined that ubiquitination but not proteasome activity is crucial for DENV RNA release into the cytoplasm from incoming virions. We found that capsid protein degradation is proteasome dependent but that its decay is not the cause of genome uncoating. Our observations support a model in which capsid protein degradation takes place independently of the engagement of the viral genome in viral translation. Importantly, we demonstrated that inhibition of the ubiquitin E1 activity blocks genome uncoating, resulting in a protected/inactive viral RNA retained inside either endosomes or nucleocapsids. These results provide the first insights into the DENV uncoating process and highlight a window of opportunity for viral inhibition.

In the last few years, studies on DNA and RNA viruses have uncovered unconventional modes of utilization of the host ubiquitin proteasome system for viral uncoating. Mercer and collaborators showed that vaccinia virus genome release from the core protein required proteasome activity; however, the process was ubiquitination independent. Interestingly, it was found that the core protein was already ubiquitinated inside the viral particle (20). For influenza A virus (IAV), ubiquitin was recently reported to be crucial for viral uncoating; unexpectedly, capsid protein with non-covalently attached ubiquitin chains was found in the virion (28). In addition, Su and collaborators identified the E3 ubiquitin ligase Itch as a fundamental factor for an early stage in the IAV life cycle (24). They found that the incorporation of viral ribonucleoprotein complex into endosomes in Itch knockdown cells was normal but that its subsequent release from endosomes was retarded. Here, we describe for DENV yet another way of employing the ubiquitin/proteasome system in which a nondegradative ubiquitination step is necessary to release the viral genome into the cytoplasm. By using pharmacological inhibition of the ubiquitin E1, or UBA1 silencing, DENV infection was blocked (Fig. 3). In this regard, an upregulation of UBA1 expression upon DENV infection in primary human cells has been previously noticed, supporting the idea of viral utilization and regulation of this host protein (29). Moreover, in the same study, a reduction in DENV titers was detected by UBA1 inhibition. Our observations are in agreement with the results of that previous study and provide an explanation for the reduced viral propagation reported.

The requirement of ubiquitination during flavivirus infection was previously proposed based on RNA interference screens (30). In that study, a requirement of the ubiquitin ligase CBLL1 in WNV internalization was found. The authors reported that inhibition of the proteasome activity abolished WNV infection; however, another group observed that CBLL1 depletion did not affect DENV or WNV particle internalization (31). Moreover, those authors claimed that neither the presence of MG132 nor that of lactacystin inhibited WNV genome entry, suggesting that the proteasome system was not required for early stages of infection (31). Our observations using DENV support the idea that MG132 does not affect viral internalization, uncoating, or early viral translation (Fig. 2). Moreover, by silencing or inhibiting the activity of the CRL family of E3 ligases, we excluded the requirement of this large group of ligases for DENV genome release (Fig. 3). Although differences between DENV and WNV infections have been noticed, it will be important to examine whether early steps in WNV infection also require a ubiquitination step that is independent of the proteasome activity.

Initially, we hypothesized that the incoming capsid protein could be subjected to ubiquitination as a signal for uncoating. In this regard, it has been reported that the host valosin-containing protein (VCP) (p97) disassembles ribonucleoprotein complexes by detecting ubiquitinated proteins (27). On the basis of this possibility, we attempted to detect ubiquitination of the capsid protein that enters the cell in virions. Although we used different assays, the analysis was hampered by the small amounts of capsid involved and the narrow window of time for detecting the incoming protein. Thus, as an alternative strategy, we developed recombinant dengue viruses with Lys replaced by Arg in the capsid protein. This strategy was complicated due to the presence of *cis*-acting RNA replication elements in the capsid coding sequence. To overcome this problem, we used a reporter DENV with duplicated capsid coding regions to uncouple RNA genome replication and virus production. Using this system, we generated a virus with a K-less capsid that, although less efficient than the WT, was able to produce infectious particles (Fig. 4). The fact that a DENV bearing a K-less capsid is able to infect cells suggests that capsid is not the target for the ubiquitination required for uncoating.

Our results show that the incoming viral RNA increased its half-life from 3 h to more than 10 h in the presence of the ubiquitin E1 inhibitor, supporting the idea that ubiquitination was necessary for genome release into the cytoplasm. Considering that the ubiquitin inhibitor does not impair viral entry and that its effect on translation was observed only if it was added at up to 30 min after infection, we propose that ubiquitination is necessary during endosome maturation, membrane fusion, or nucleocapsid disassembly (Fig. 5). Thus, we conclude that ubiquitination is required postentry and pretranslation.

For different viruses, interference with capsid protein functions can greatly disturb genome release during viral entry, abolishing viral infection. This explains why the capsid proteins of enveloped viruses have been regarded as attractive targets for the design of a new generation of antiviral agents in recent years (32). Recently, ST-148, a small molecule that potently inhibits *in vitro* replication of all four serotypes of DENV, was identified (9). The characterization of the mode of action of ST-148 showed that it enhances capsid protein self-interaction, perturbing assembly and disassembly of nucleocapsids (10). Importantly, the assays performed in tissue culture and in infected mice exhibited no selection of ST-148-resistant viruses despite the presence of drug-resistant variants in the population (11). The hypothesis of genetic dominance of defective subunits proposes that oligomerization of capsid units that include both resistant and susceptible proteins in infected cells would generate nonfunctional complexes, causing a delay in the emergence of resistant viruses (11). Thus, targeting capsid functions is emerging as an attractive goal for developing antivirals to control DENV infection.

Taking the results together, our work has provided new information about the fate of viral nucleocapsid components during DENV infection and has defined the requirement of ubiquitination, independently of the proteasome activity, for genome uncoating. Hopefully, a better understanding of the host-virus interaction and the viral processes at the molecular level will provide the necessary tools for DENV control.

## MATERIALS AND METHODS

### Cell lines and viruses.

Baby hamster kidney cells (BHK-21) were cultured in minimum essential medium alpha supplemented with 10% fetal bovine serum. Human lung cell line (A549) cells were cultured in Dulbecco’s modified Eagle’s medium–Ham F-12 medium supplemented with 10% fetal bovine serum. C6/36 HT mosquito cells from *Aedes albopictus*, adapted to grow at 33°C, were cultured in L-15 medium (Leibovitz) supplemented with 0.3% tryptose phosphate broth, 0.02% glutamine, 1% modified Eagle’s medium (MEM) nonessential amino acid solution, and 10% fetal bovine serum. All media were supplemented with 100 U/ml penicillin and 100 µg/ml streptomycin. Stocks of DENV serotype 2 16681 were prepared in mosquito C6/36 cells and used to infect the different cell lines as indicated in each case.

### Construction of recombinant DENVs.

Dengue virus mutants were generated using a DENV type 2 cDNA clone (pD2/IC) (33), with an additional AflII restriction site just upstream of the polyprotein stop codon and a NotI restriction site at nucleotide 244 (pD2/ICAflII-NotI). Mutations were introduced into the full-length cDNA of DENV 2 pD2/IC AflII, replacing the SacI/NotI or NotI/SphI fragment of the WT plasmid with a fragment derived from overlapping PCR mixtures containing the desired mutation as previously described (5). For reporter virus studies, the mutations were introduced in the context of the DV-R virus (14).

### RNA transcriptions and transfections.

DENV genomic RNA was obtained by *in vitro* transcription using T7 RNA polymerase in the presence of an m7GpppA cap analog. The corresponding plasmids were linearized with XbaI and were purified by phenol-chloroform extraction. RNA integrity was confirmed using 1% agarose gels. RNA transfections were performed with Lipofectamine 2000 (Invitrogen) according to the manufacturer’s instructions. For reporter DENVs, 500 ng of RNA transcripts was transfected into BHK-21, A549, or C6/36 HT cells grown in 24-well plates. The *Renilla* luciferase activity present in cell extracts was analyzed at the times indicated in the figures, according to the manufacturer’s instructions (Promega). The supernatants collected at the times indicated in the figures were stored at −80°C. For indirect immunofluorescence (IF) assays, 500 ng of RNA transcripts was transfected into BHK-21 cells grown in 35-mm-diameter tissue culture dishes.

### Immunofluorescence assays.

Transfected BHK-21 cells with WT or mutated DENV RNAs were grown in 35-mm-diameter tissue culture dishes containing 1-cm^2^ coverslips. At various times posttransfection, the coverslips were removed, and the cells were fixed with methanol for 15 min at −20°C. To maintain cell viability for a long time, cells were trypsinized every 3 days, and a one-third volume of the total cells and the supernatant was reseeded in a 35-mm-diameter tissue culture dish containing a new coverslip. For the detection of viral antigens, a specific anti-E monoclonal antibody (MAb) E18 (34) was used. Alexa Fluor 488-conjugated rabbit anti-mouse immunoglobulin G (Molecular Probes) was employed to detect the primary antibody under the same conditions.

### Inhibitors.

The compound UBEI 41 (Pyr-41), a cell-permeable inhibitor of ubiquitin-activating enzyme (E1), was purchased from Sigma-Aldrich, as well as the proteasome inhibitor MG-132. The concentrations employed for assays were 75 µM and 20 µM, respectively. Cycloheximide (Sigma-Aldrich) was used at a concentration of 75 µg/ml.

### Western blotting.

A549 cells infected with WT DENV2 were grown in 35-mm-diameter tissue culture dishes. For the detection of capsid protein in A549 cell extracts, cells were harvested in each experiment at the time indicated, washed with phosphate-buffered saline (PBS), and treated (when specified) with proteinase K (1 mg/ml) for 45 min at 4°C. The proteinase K was inactivated (2 mM phenylmethylsulfonyl fluoride [PMSF]–PBS–3% bovine serum albumin [BSA]), and the cells were lysed using buffer H (10 mM HEPES [pH 7.9], 50 mM KCl, 2 mM EDTA, 0.5 mM PMSF, 1 mM dithiothreitol [DTT], 0.5 mM Triton X-100). Samples were analyzed by 15% SDS-polyacrylamide gel electrophoresis (SDS-PAGE) under denaturing conditions, and Western blotting was performed using a specific rabbit anti-C polyclonal antibody obtained in our laboratory as described previously (14).

### RNA interference.

RNA interference experiments were carried out using ON-TARGETplus SMARTpool siRNA oligonucleotides (Dharmacon RNA Technologies). RNA interference (RNA) was directed to UBA1 (7317) and UBA3 (9039). The control used was directed to *Renilla* luciferase and the unrelated siRNA to Firefly luciferase. After 24 h, the reaction mixtures were seeded in 24-well plates and A549 cells were transfected with the corresponding siRNA using Oligofectamine (Invitrogen). Briefly, 25 pmol of siRNA and 50 µl of Opti-MEM (Invitrogen) were mixed with 2 µl of Oligofectamine in 50 µl of Opti-MEM and incubated for 20 min. The mix was added to a monolayer of 50%-confluent A549 cells and incubated overnight. Then, the medium was replaced with complete Dulbecco’s modified Eagle’s medium (DMEM)-F12 medium. After 48 h of transfection, cells were infected with DV-R and luciferase levels were measured at 6 or 48 h.

### RNA extraction, reverse transcription, and real-time PCR.

For the quantification of viral RNA by real-time reverse transcriptase PCR (RT-PCR), the RNA from cells infected with DENV and treated with proteinase K was extracted with TRIzol (Invitrogen) at various times postinfection. RNA extracted from experimental samples was reverse transcribed in 20-µl reaction volumes using 160 units of Moloney murine leukemia virus reverse transcriptase (M-MLV RT) (Promega), 4 units of RNasin RNase inhibitors (Promega), 500 nM primer A.V.G. 572 (5′ ACCAAGGACTCCTGCCTCTTCC 3′), 0.5 mM (each) deoxynucleoside triphosphates (dNTPs), and 1× M-MLV RT buffer. Primer and RNA template were preincubated for 5 min at 70°C. The RT reaction was allowed to proceed for 1 h at 42°C and then for 15 min at 65°C to denature the RT enzyme. For real-time quantitative PCR (qPCR), an Mx3005P QPCR system (Agilent Technologies Inc.) was employed. Reactions were performed in triplicate in 96-well plates using 2 µl of the RT reaction mixture as the template (and a 1:10 dilution in RNase-free water), 5 µl of FastStart Universal SYBR green Master (Rox) 2× mix (Roche), 300 nM (each) primer (5′ GAATACACAGATTACATGCC 3′ and A.V.G. 572), and RNase-free water (added to reach a volume of 10 µl). Reactions were run using the following parameters: 95°C for 15 min and 40 repeats of 95°C for 10 s and 60°C for 30 s. Fluorescence detection was acquired during the elongation step at each cycle. Regarding the UBA1 and UBA3 qPCRs, the primers used for amplification were UBA1 Fw (5′ GATTTCATCGTGGCTGCATCCAAC 3′) and Rev (5′ CTTGTAGGAGTCAAGCTGTCGGT 3′) and UBA3 Fw (5′ GATTTCATCGTGGCTGCATCCAAC 3′) and Rev (5′ CTACCCCTTGAGTGAGCCTATAC 3′). The RT reaction was performed with hexa-random primers.
